# Supplementary material for: Fibrochondrogenic potential of synoviocytes from osteoarthritic and normal joints cultured as tensioned bioscaffolds for meniscal tissue engineering in dogs
Source: PeerJ. 2014 Sep 30;2:e581. doi: 10.7717/peerj.581 (PMC4183955; doi:10.7717/peerj.581)
Supplement: Supplemental Information 2 [file peerj-02-581-s002.doc]

# Informed Consent Form for Animal Owners

## Project Title: Canine Meniscal Tissue Engineering

## Principal Investigator: Jennifer Warnock DVM PhD DACVS, Department of Clinical Sciences

## Co-Investigators: Wendy Baltzer, DVM, PhD, DACVS, Department of Clinical Sciences

# **PURPOSE OF STUDY:**

- **In canine knee surgery certain diseased tissues are routinely cleaned out of the joint and discarded. This includes torn meniscus tissue, inflamed fat, and over-abundant joint lining (synovium).**
- **The purpose of this study is to keep these tissues which are normally discarded for *in vitro* cell culture, with the long term purpose of building new joint structures such meniscus in the laboratory.**

**PURPOSE OF THE FORM:**

- You are being asked to give your consent to have your dog (*Canis familiaris*) participate in a research study. This consent form gives you the information you will need to help you decide whether to allow your animal to participate.
- Please read the form carefully.
- You may ask any questions about the research, the possible risks and benefits, rights as a volunteer participant, and anything else that is not clear.
- When all of your questions have been answered, you can decide if you will allow your animal to be in this study or not.

WHAT WILL HAPPEN IN THE STUDY:

- During normal knee surgery, torn ligament, torn meniscus, fat, and joint lining is cleaned out of the joint and discarded. Instead of disposing these tissues, they will be kept and cultured in the lab.
- With proper growing techniques we hope to turn damaged tissue into useful joint structures.

**RISKS OF THE STUDY:**

- Your dog may have been referred to the OSU VMTH for a knee surgery consultation. This study does not change the way we treat knee diseases in dogs, but it does change the way tissues are handled after they are removed from the joint. You pet’s need for surgery and the possible complications of that surgery are independent of this study, and will be discussed by your surgeon.

**BENEFITS OF THE STUDY:**

- If these long term studies are successful, we may potentially have replacement tissue waiting for your pet in the lab should the need arise!
- If you have personal interest in the field of tissue engineering, we can email you updates on the cells as they grow in the lab.

**IS THERE COMPENSATION FOR PARTICPATING:**

 No.

**ARE THERE ANY COSTS FOR PARTICIPATING:**

 No.

**IN CASE OF INJURY:**

Your surgeon will discuss all possible surgical complications associated with your pet’s knee surgery. You surgeon can also be reached at any time of the day through our normal business phone line and after hours emergency service.

**WITHDRAWING MY ANIMAL FROM THE STUDY:**

Your participation in this study is entirely voluntary and you may withdraw your animal at any time. You also understand that your animal may be withdrawn from the study if the investigators find it necessary. If your animal is withdrawn from the study for any reason, data already collected may continue to be used for research purposes.

You will not be treated differently if you decide to not participate in this study. Your decision to participate, not participate, or withdraw from the study will not affect your relationship with OSU.

**WHAT IF I HAVE QUESTIONS?**

If you have any questions about the study, you may contact Dr. Warnock at (541)-737- 6859, or jennifer.warnock@oregonstate.edu.

Your signature indicates that this research study has been explained to you, that your questions have been answered and that you agree to allow your animal to be in the study. You will receive a copy of this form.

Animal’s Name:_______________________________

Owner’s Printed Name:_____________________________

Owner’s Signature: _________________________________ Date:____________

Principal Investigator’s Printed Name:__Jennifer Warnock DVM_________________

Principal Investigator’s Signature: _______________________________

Date: ____________

I would like to know how my pet’s cells are doing in culture:

_____Yes _________No

My email address is: _________________________________________-
