# Supplementary material for: Fibrochondrogenic potential of synoviocytes from osteoarthritic and normal joints cultured as tensioned bioscaffolds for meniscal tissue engineering in dogs
Source: PeerJ. 2014 Sep 30;2:e581. doi: 10.7717/peerj.581 (PMC4183955; doi:10.7717/peerj.581)
Supplement: Supplemental Information 3 [file peerj-02-581-s003.pdf]

Institutional Animal Care and Use Committee ▪ Office of Research Integrity
Oregon State University, 308 Kerr Administration Building, Corvallis, Oregon 97331-2140
Tel 541-737-2762 | **Fax** 541-737-3093 | http://oregonstate.edu/research/ori/index.htm
IACUC@oregonstate.edu

May 1, 2009

Dr. Warnock,

The IACUC staff reviewed a proposal from you ("Canine Cell Culture for In Vitro Chondrogenesis and Fibrochondrogenesis"), and determined that the proposed word does not require IACUC review and approval. The proposal described the use of tissue collected during operations being performed in the Small Animal Clinic of the Veterinary Teaching Hospital to repair dog's knee(s). As described, these surgeries and the removal of various joint tissue would be performed regardless of your proposed research. Therefore, the proposed work does not constitute regulated animal use, and there is no need for IACUC approval. If the tissue would be removed specifically for the project involved, IACUC approval would be required, in advance.

Should there be need to perform manipulations with animals in the future, such as requesting removal of tissues not a by product of a normal knee repair operation or alterations of the normal repair surgery (e.g., removing more tissue, other tissues, etc.), then IACUC approval should be sought in advance of any manipulations.

It is understood that your tissue culture work may lead to the need to manipulate live animals in the future. This type of work would require advance IACUC review and approval.

This letter serves to document that obtaining tissue that would otherwise be disposed does not require IACUC approval. If there is a need to change this use, please contact the IACUC Office, 541-737-2762, in advance to determine if review and approval are needed.

Sincerely,

Dr. Andrew Buermeyer
IACUC Chair
