# Supplementary material for: Fibrochondrogenic potential of synoviocytes from osteoarthritic and normal joints cultured as tensioned bioscaffolds for meniscal tissue engineering in dogs
Source: PeerJ. 2014 Sep 30;2:e581. doi: 10.7717/peerj.581 (PMC4183955; doi:10.7717/peerj.581)
Supplement: Appendix S1 — Assays used for RT-PCR [file peerj-02-581-s004.docx]

Appendix 1: Assays used for RT-PCR

| ***Gene*** | ***Amplicon***  ***Size*** | ***Assay Catalog Number*** | ***Reference Sequence*** |
| --- | --- | --- | --- |
| Interleukin 1, beta | 70 | Cf02671952_m1 | NM_001037971.1 |
| Interleukin 6 | 68 | Cf02624151_m1 | NM_001003301.1 |
| Tumor Necrosis Factor-alpha | 131 | Cf02628237_m1 | NM_001003244.4 |
| SOX-9 | 103 | Cf02625134_g1 | NM_001002978.1 |
| Collagen, Type I, alpha 1 | 87 | Cf02623126_m1 | NM_001003090.1 |
| Collagen, Type II, alpha 1 | 89 | Cf02622862_m1 | NM_001006951.1 |
| Aggrecan | 125 | Cf02674826_m1 | NM_001113455.1 |
| GAPDH | 97 | AIWRF9W | NM_001003142.1 |
